# Supplementary figures and images for: Intrinsic anti-Stokes emission in living HeLa cells
Source: PLoS One. 2020 Mar 16;15(3):e0230441. doi: 10.1371/journal.pone.0230441 (PMC7075565; doi:10.1371/journal.pone.0230441)

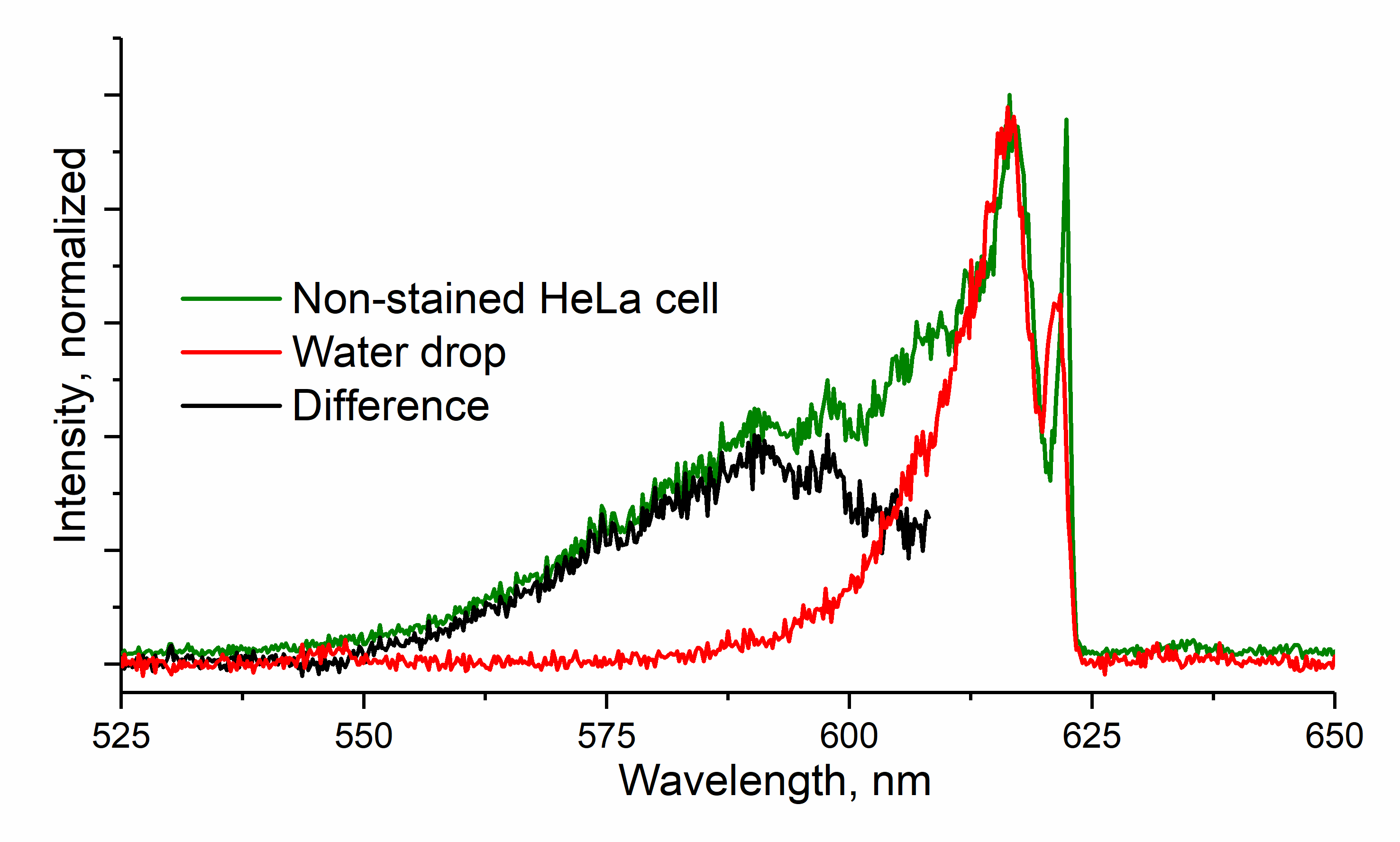

Supplement: S1 Fig — (TIF) [file pone.0230441.s001.tif]

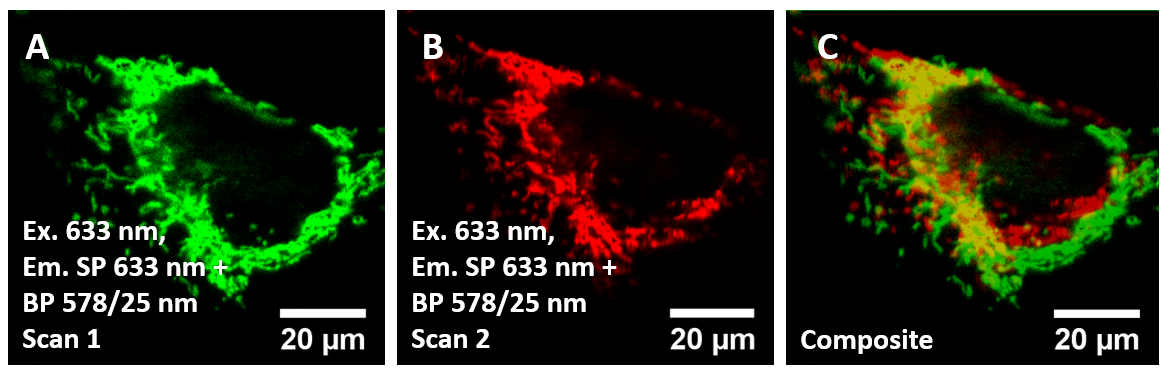

Supplement: S2 Fig — Two consecutive anti-Stokes emission scans (A and B) and their composite (C) (measured on non-stained HeLa cells, ~10 min delay between the scans), showing differences between the images arising due to cell movement. (TIF) [file pone.0230441.s002.tif]

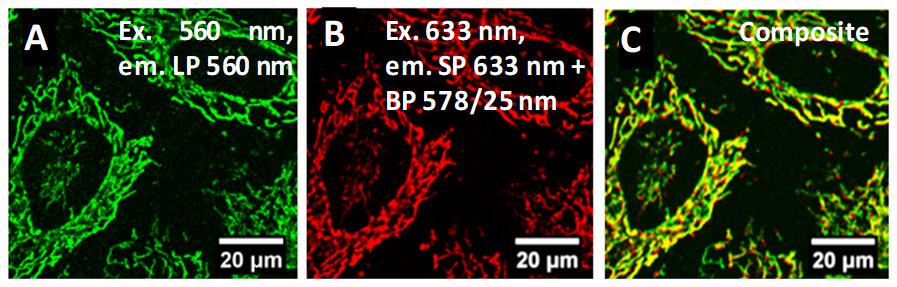

Supplement: S3 Fig — Auto-fluorescence (A), anti-Stokes emission (B) and composite (C) images of MitoTracker-Green stained HeLa cells. Note that at 560 nm MitoTracker-Green does not absorb and we are monitoring the intrinsic auto-fluorescence. (TIF) [file pone.0230441.s003.tif]

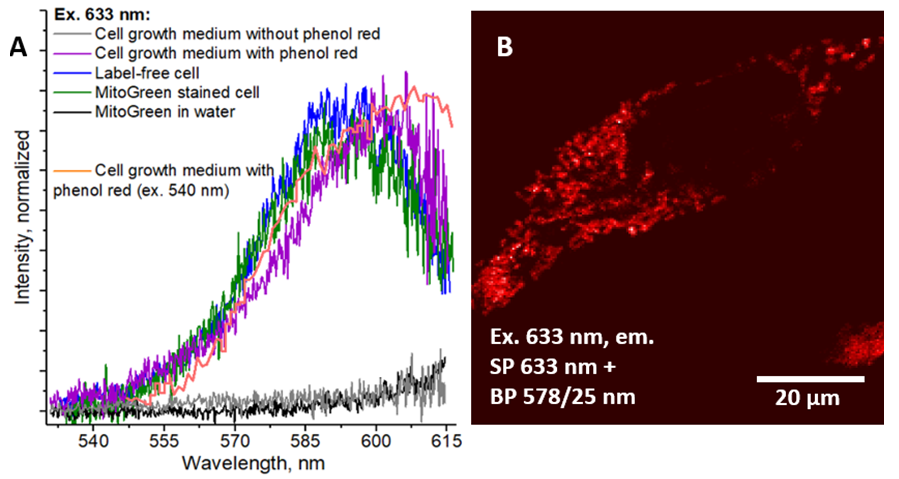

Supplement: S4 Fig — Normalized anti-Stokes emission spectra (ex. 633 nm) of various samples compared to Stokes fluorescence (ex. 560 nm, orange spectrum) of phenol red (A). Cells here were grown in phenol red containing medium and exchanged to phenol red-free medium before imaging. Label-free HeLa cells, cultivated in phenol red-free medium for 4 weeks (B). (TIF) [file pone.0230441.s004.tif]

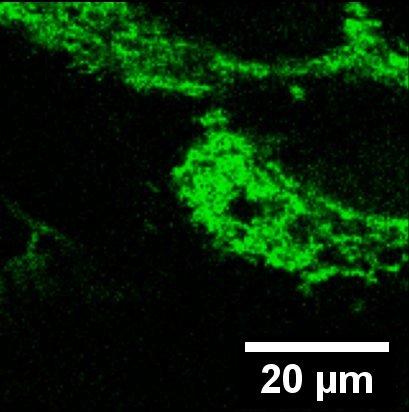

Supplement: S1 Datasets — (ZIP) [file pone.0230441.s005.zip › Data/Fig 1/A.jpg]

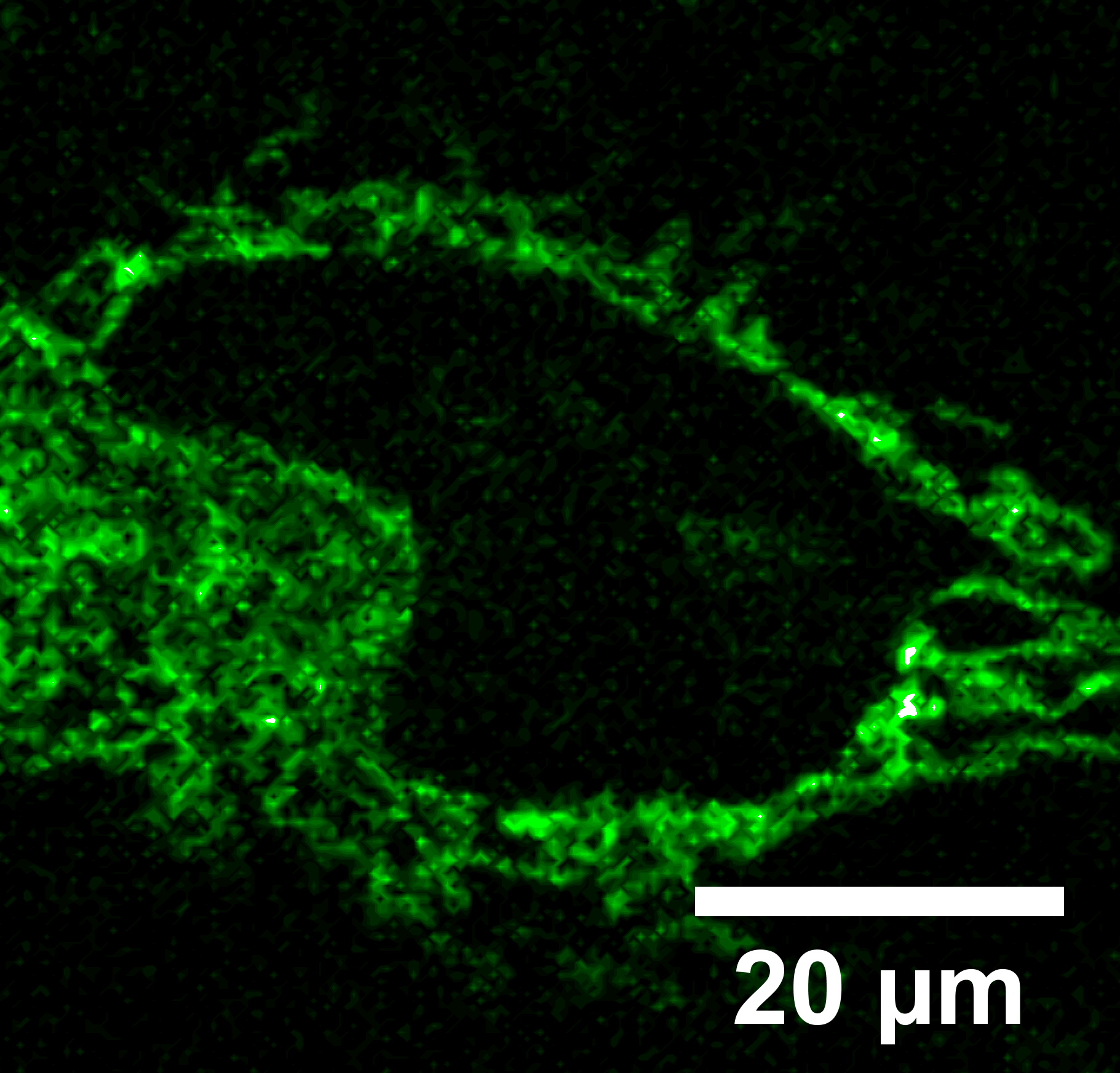

Supplement: S1 Datasets — (ZIP) [file pone.0230441.s005.zip › Data/Fig 1/B.tif]

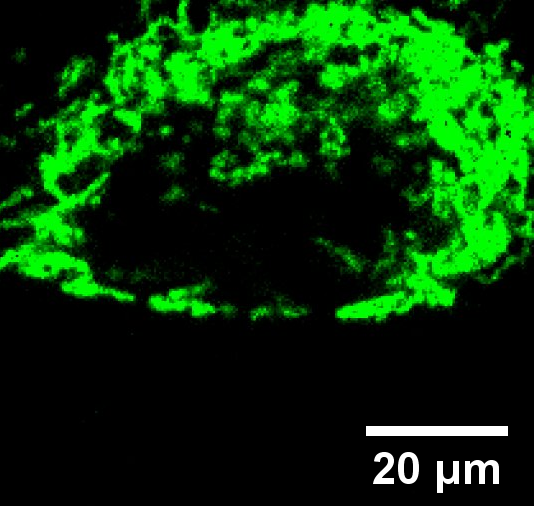

Supplement: S1 Datasets — (ZIP) [file pone.0230441.s005.zip › Data/Fig 1/C.png]

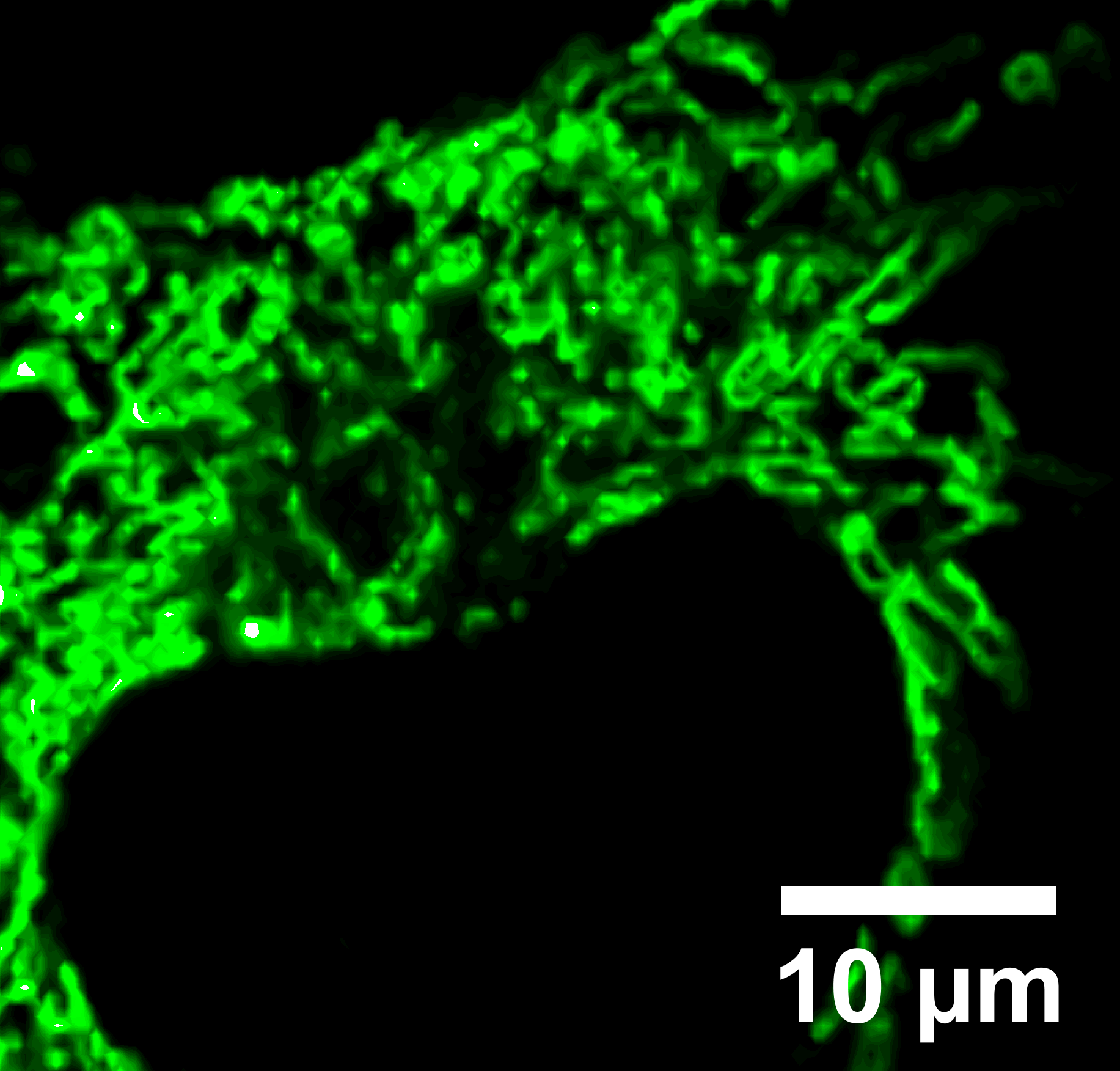

Supplement: S1 Datasets — (ZIP) [file pone.0230441.s005.zip › Data/Fig 1/D.tif]

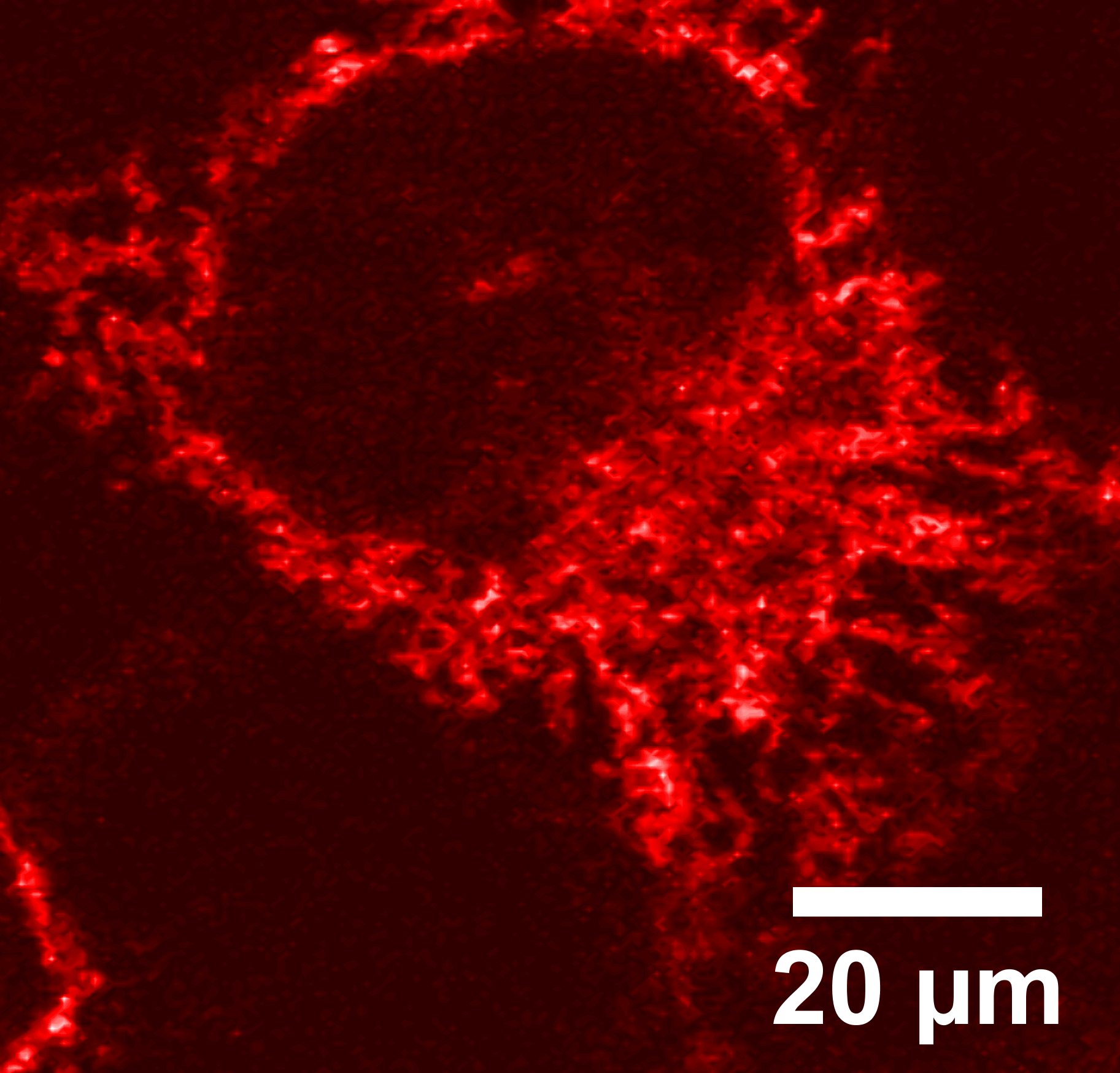

Supplement: S1 Datasets — (ZIP) [file pone.0230441.s005.zip › Data/Fig 1/E.tif]

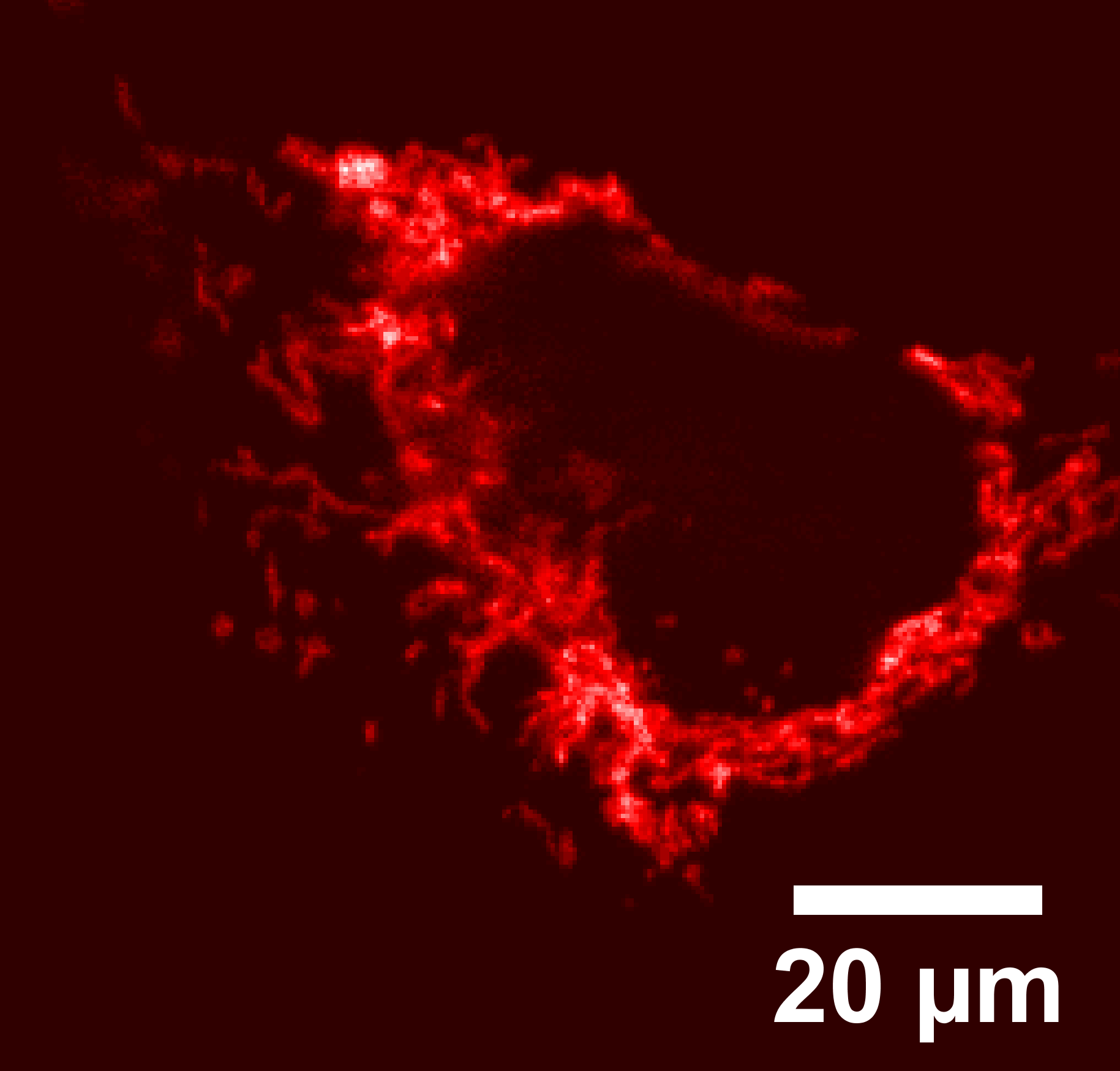

Supplement: S1 Datasets — (ZIP) [file pone.0230441.s005.zip › Data/Fig 1/F.tif]

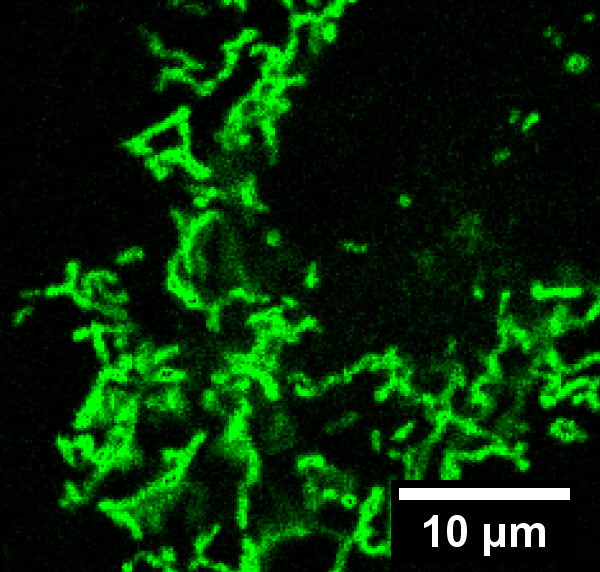

Supplement: S1 Datasets — (ZIP) [file pone.0230441.s005.zip › Data/Fig 2/A.png]

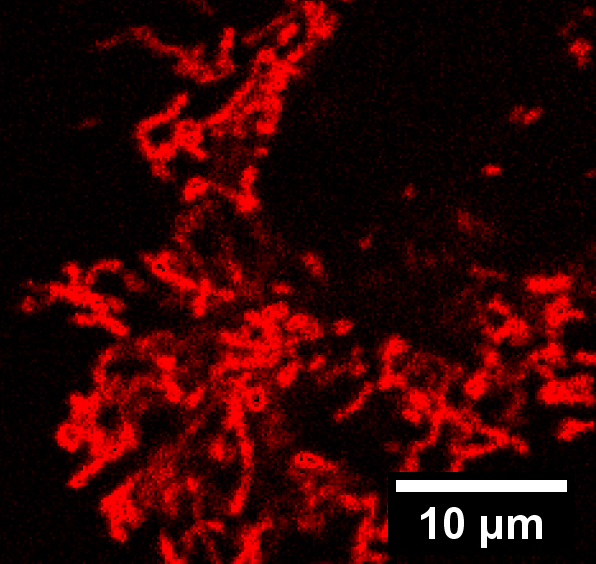

Supplement: S1 Datasets — (ZIP) [file pone.0230441.s005.zip › Data/Fig 2/B.png]

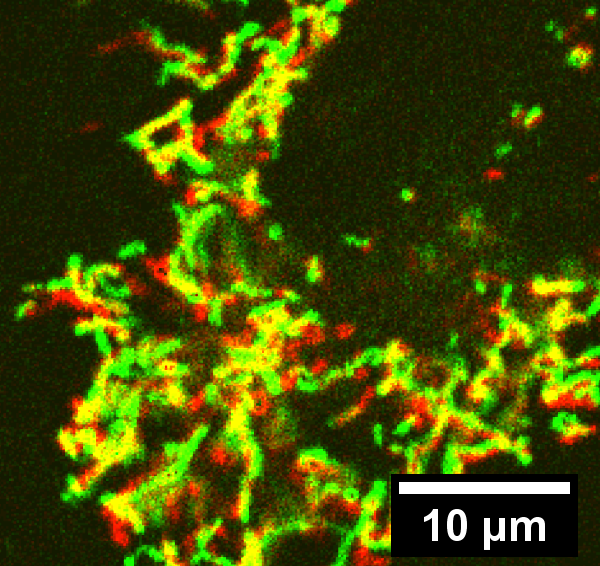

Supplement: S1 Datasets — (ZIP) [file pone.0230441.s005.zip › Data/Fig 2/C.png]

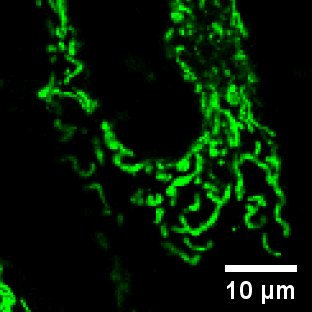

Supplement: S1 Datasets — (ZIP) [file pone.0230441.s005.zip › Data/Fig 2/D.jpg]

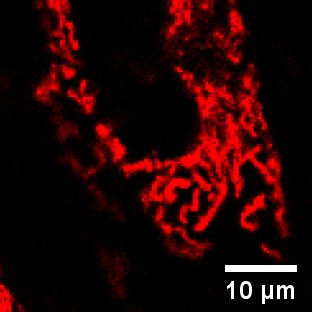

Supplement: S1 Datasets — (ZIP) [file pone.0230441.s005.zip › Data/Fig 2/E.jpg]

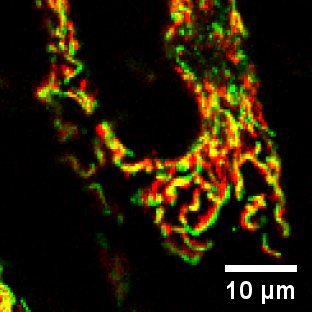

Supplement: S1 Datasets — (ZIP) [file pone.0230441.s005.zip › Data/Fig 2/F.jpg]

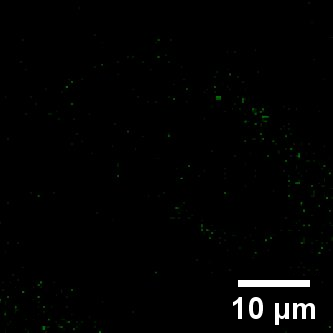

Supplement: S1 Datasets — (ZIP) [file pone.0230441.s005.zip › Data/Fig 3/B.tif]

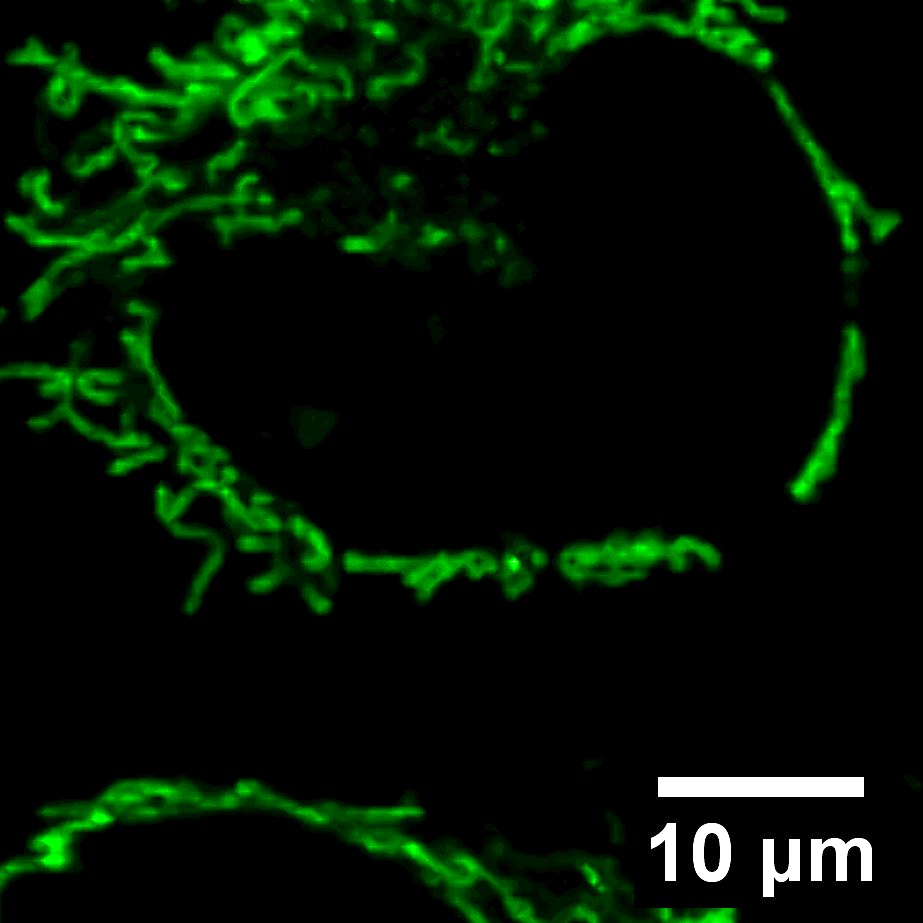

Supplement: S1 Datasets — (ZIP) [file pone.0230441.s005.zip › Data/Fig 3/D.tif]

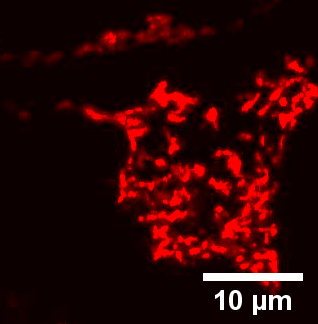

Supplement: S1 Datasets — (ZIP) [file pone.0230441.s005.zip › Data/Fig 4/A.tif]

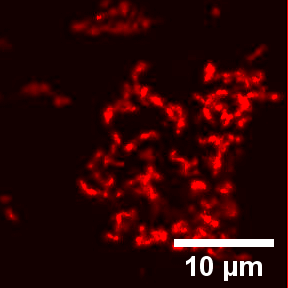

Supplement: S1 Datasets — (ZIP) [file pone.0230441.s005.zip › Data/Fig 4/B.tif]

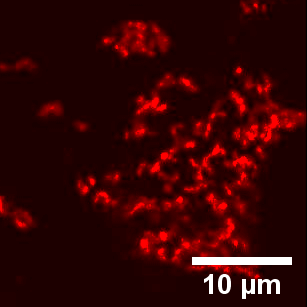

Supplement: S1 Datasets — (ZIP) [file pone.0230441.s005.zip › Data/Fig 4/C.tif]

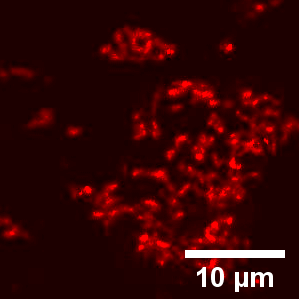

Supplement: S1 Datasets — (ZIP) [file pone.0230441.s005.zip › Data/Fig 4/D.tif]

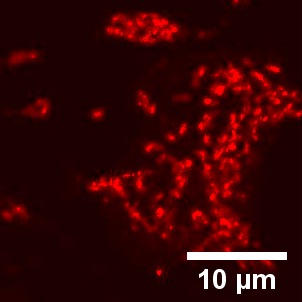

Supplement: S1 Datasets — (ZIP) [file pone.0230441.s005.zip › Data/Fig 4/E.tif]

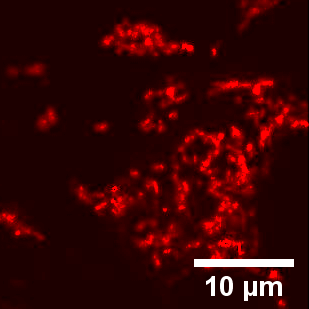

Supplement: S1 Datasets — (ZIP) [file pone.0230441.s005.zip › Data/Fig 4/F.tif]

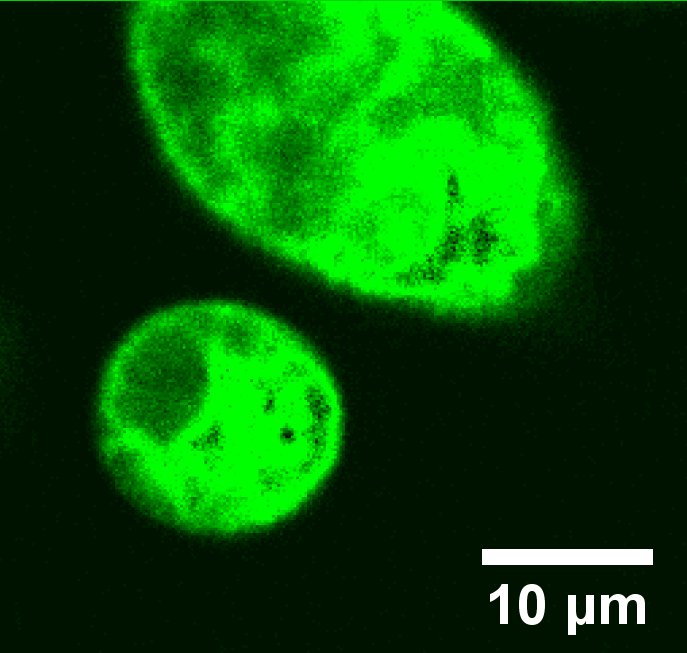

Supplement: S1 Datasets — (ZIP) [file pone.0230441.s005.zip › Data/Fig 4/G.jpg]

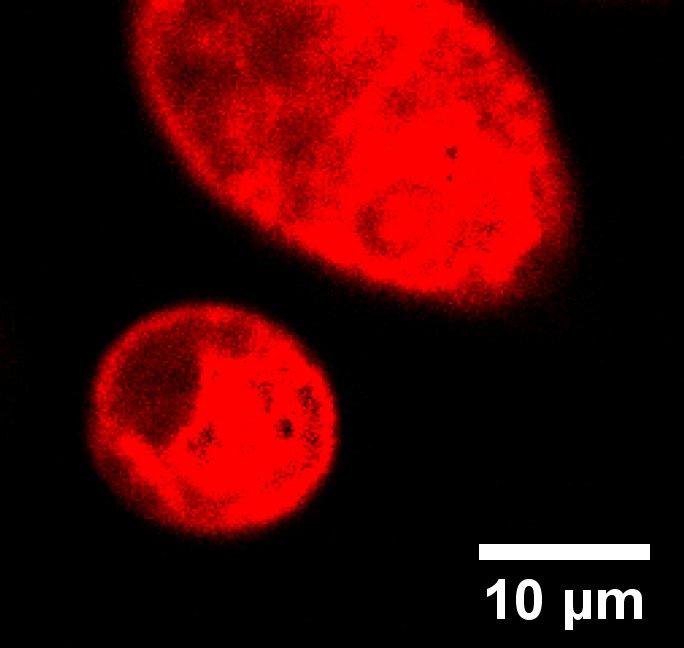

Supplement: S1 Datasets — (ZIP) [file pone.0230441.s005.zip › Data/Fig 4/H.jpg]

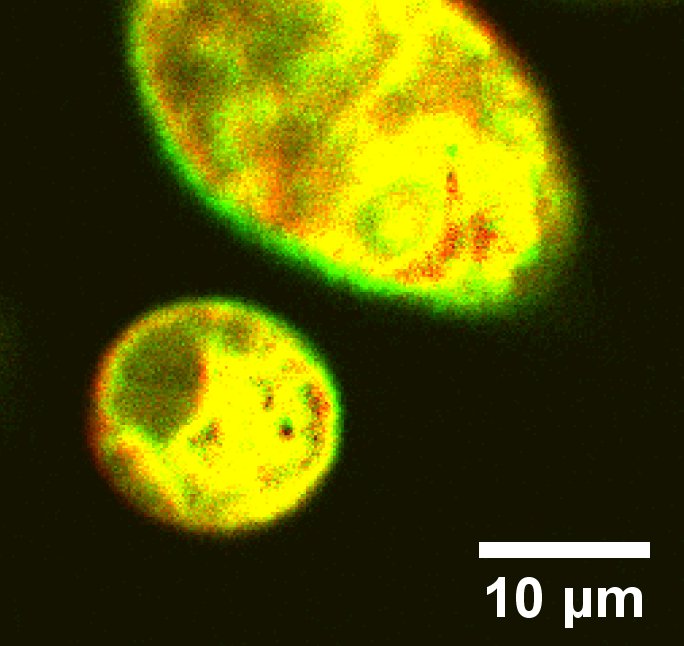

Supplement: S1 Datasets — (ZIP) [file pone.0230441.s005.zip › Data/Fig 4/I.jpg]

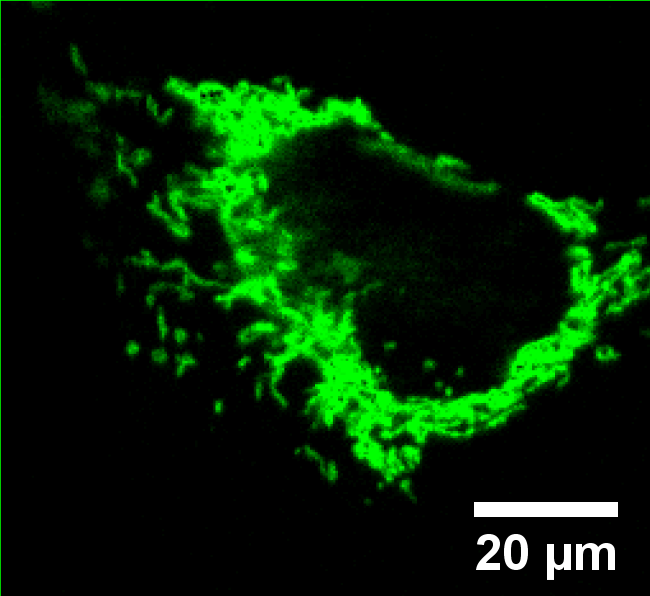

Supplement: S1 Datasets — (ZIP) [file pone.0230441.s005.zip › Data/Fig SI 2/A.tif]

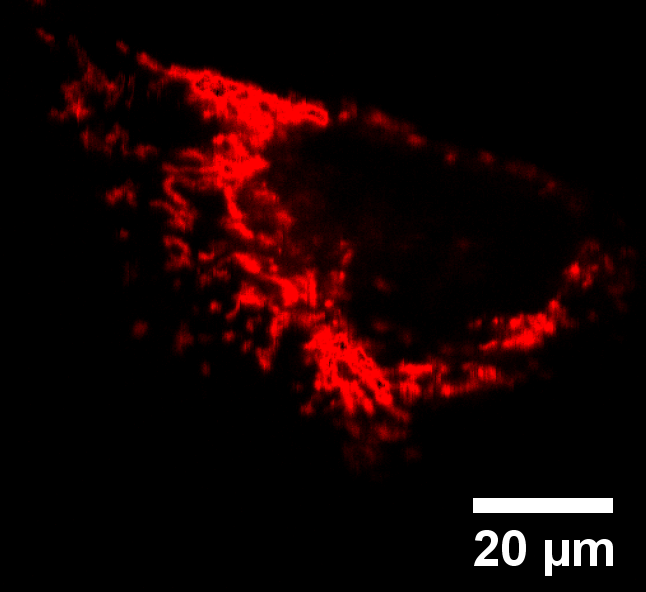

Supplement: S1 Datasets — (ZIP) [file pone.0230441.s005.zip › Data/Fig SI 2/B.tif]

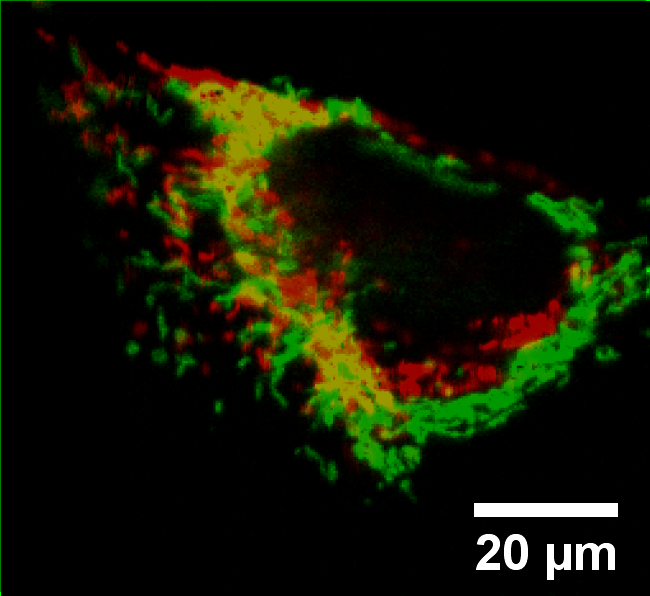

Supplement: S1 Datasets — (ZIP) [file pone.0230441.s005.zip › Data/Fig SI 2/C.tif]

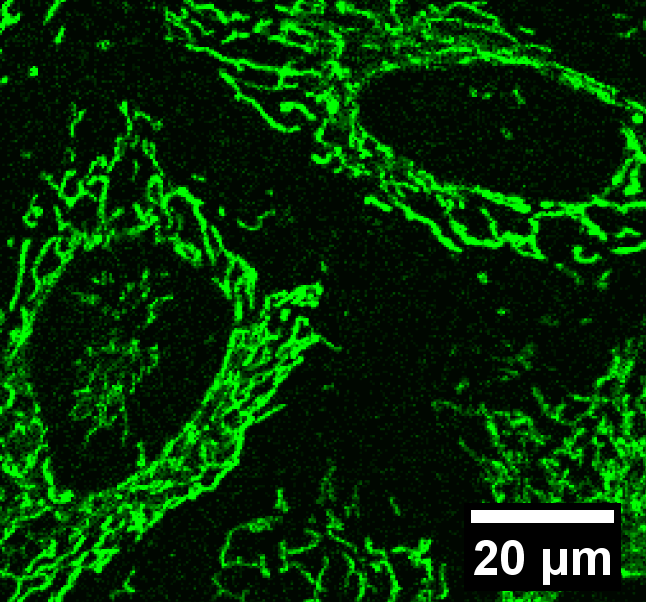

Supplement: S1 Datasets — (ZIP) [file pone.0230441.s005.zip › Data/Fig SI 3/A.png]

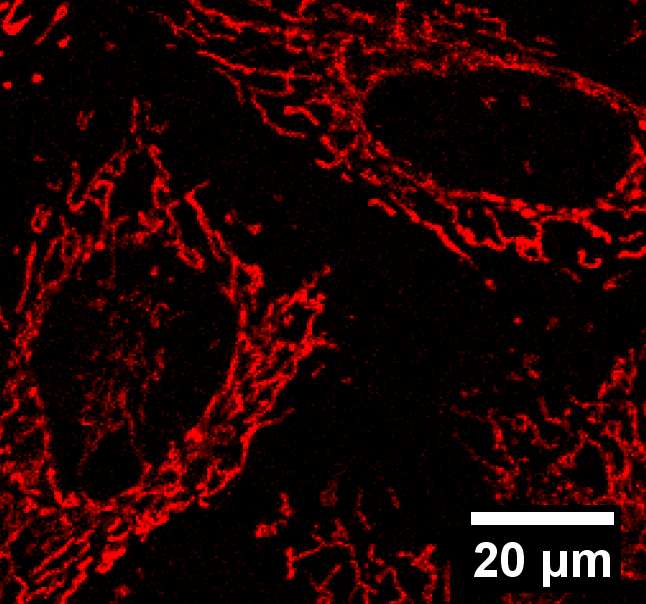

Supplement: S1 Datasets — (ZIP) [file pone.0230441.s005.zip › Data/Fig SI 3/B.png]

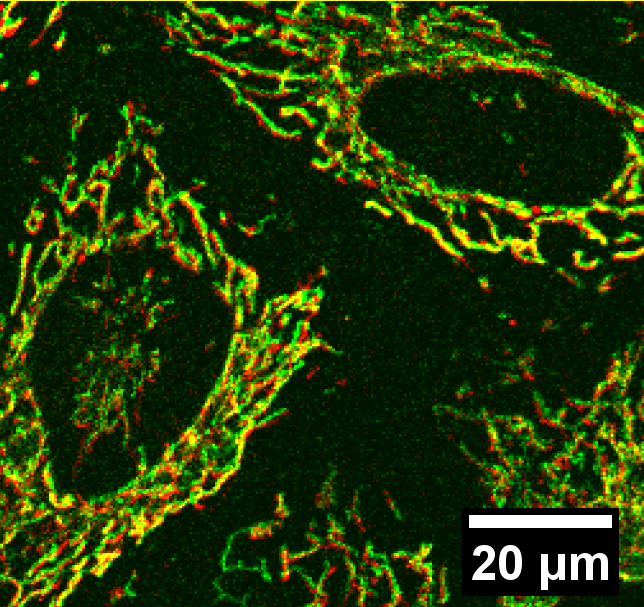

Supplement: S1 Datasets — (ZIP) [file pone.0230441.s005.zip › Data/Fig SI 3/C.png]

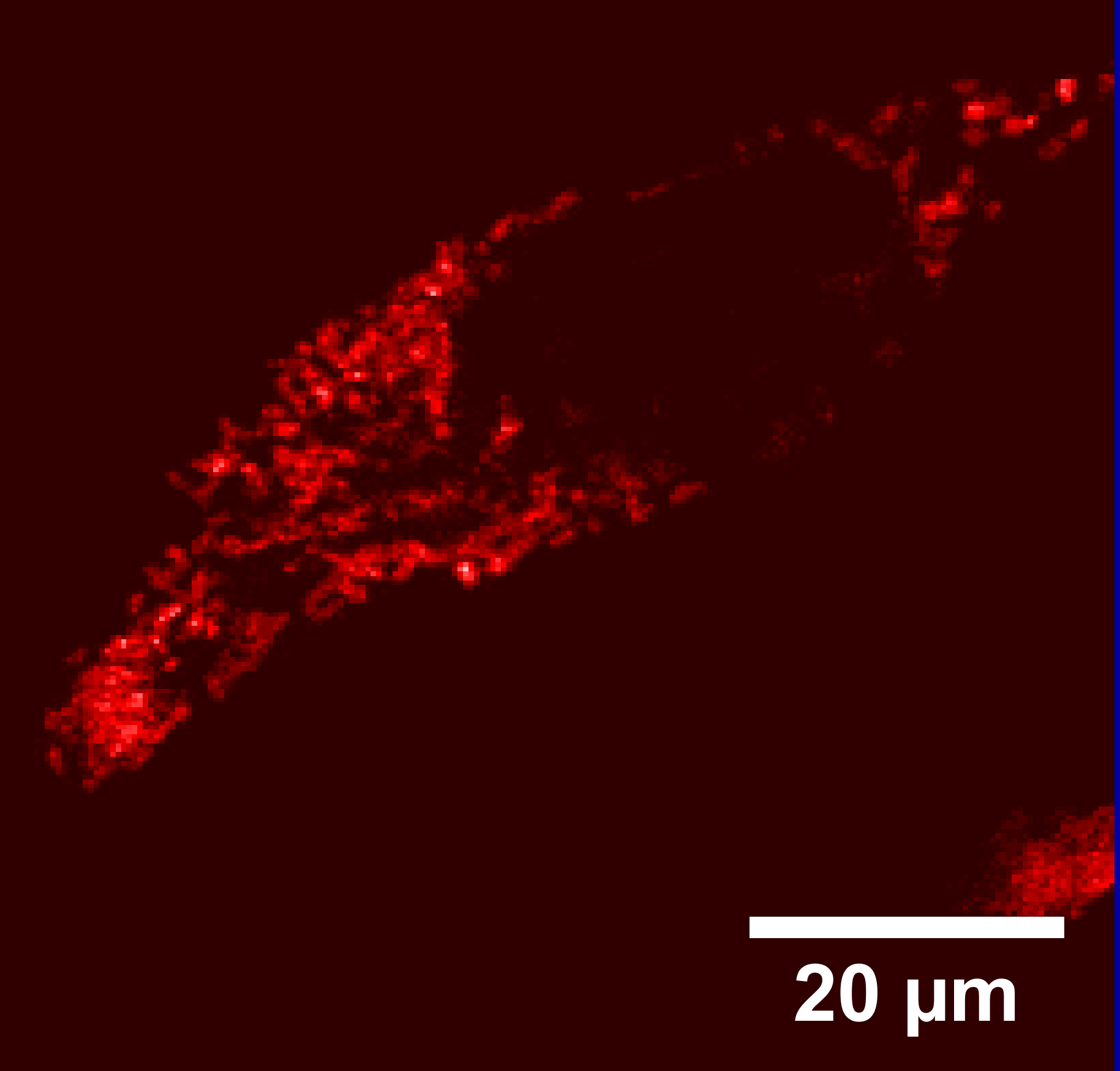

Supplement: S1 Datasets — (ZIP) [file pone.0230441.s005.zip › Data/Fig SI 4/B.tif]
