# Supplementary material for: Intrinsic anti-Stokes emission in living HeLa cells
Source: PLoS One. 2020 Mar 16;15(3):e0230441. doi: 10.1371/journal.pone.0230441 (PMC7075565; doi:10.1371/journal.pone.0230441)
Supplement: Attachment — Submitted filename: Response to Reviewers.docx [file pone.0230441.s006.docx]

**November 12^th^ 2019**

**Laura Kacenauskaite**

PhD fellow

Nano-Science Center &

Department of Chemistry

University of Copenhagen

Universitetsparken 5

DK-2100 Copenhagen Ø

Denmark

Tel: +45 71420031

laura.kacenauskaite@chem.ku.dk

f

Dear Dr Debabrata Goswami and editorial board of *PLOSONE*,

please find attached our answers to the reviewers regarding the manuscript entitled:

**Intrinsic anti-Stokes emission in living HeLa cells,**

*by Laura Kacenauskaite, Dovydas Gabrielaitis, Nicolai Bærentsen, Karen L. Martinez, Tom Vosch and Bo W. Laursen*

We addressed all reviewer comments in detail. Our corresponding changes in manuscript are marked in yellow, whereas parts where reviewer comments have been addressed are marked in blue.

We hope that with these changes the manuscript can be accepted for publication.

Best regards,

Laura Kacenauskaite

Reply to reviewers

**Reviewer 1**:

*The paper is coherent and describes an advancement in imaging techniques. The following minor grammar corrections are suggested:
- 31: "Concentration, distribution" -> "The concentration distribution"
- 38: "autofluorophores do not" -> "autofuorophores*

We are thankful for comments and positive feedback. We included these suggestions in a revised manuscript.

**Reviewer 2**:

*The manuscript titled ‘Intrinsic anti-Stokes emission in living HeLa cells’ presents an observation of anti-stokes yellow emission from the mitochondria of HeLa cells. While this was an interesting observation to start with, unfortunately the authors overlooked existing literature and failed to verify the possible source and possible underlying mechanism of this emission. The manuscript is well written and data is presented concisely.*

*1) First of all, it should be mentioned that auto-fluorescence from mitochondria is well known and extensively studied. Mitochondria is the powerhouse of a cell and host an enormous amount of electron carriers. In literature, most of the auto-fluorescence has been assigned to electron carriers like NAD, NADP, FAD etc which shuttles between various emissive states depending on their oxidation status. As the author conclusively show that the emission originates in mitochondria, it’s advisable that the authors discuss their findings in this context throughout the paper.*

We are grateful for reviewer’s comments. Stokes-side autofluorescence in multiple cell organelles, including mitochondria, is indeed well-known and studied in detail. We briefly discussed most notable examples in the first two paragraphs of the manuscript. To stress the extensive research done specifically on electron carriers in mitochondria we added more references throughout the manuscript.

However, we also want to emphasize that with this paper we are presenting completely new way of mitochondria visualization using anti-Stokes autofluorophores. We strongly believe that this fluorescence is not due to two-photon absorption (see arguments in the following comment/answer), in contrary to majority of papers published on anti-Stokes autofluorescence to this day. Therefore, we believe that more extensive review of two-photon absorption based autofluorescence microscopy could be confusing and most importantly misleading to the readers.

*2) Redox related anti-stokes emission from cellular auto-fluorescence has been reported by Melissa C. Skala and co-workers (PNAS 2007) and followed by many other groups. The 2-photon auto-fluorescence imaging is emerging as a powerful tool for optical redox imaging of cancer cells to predict malignancy. It should be noted that FAD emission occurs in the yellow region after exciting with a red laser. The fluorescence lifetime can be measured to differentiate between bound and un-bound states of these electron carriers. It’s recommended that the author to consider these literature for the best of their interest.*

We acknowledged reviewer’s recommendation and added the reference to the manuscript.

While two-photon absorption induced emission of FAD was indeed well studied by multiple research groups (including paper by Melissa C. Skala) we believe and want to stress that anti-Stokes emission reported here is not due to coherent (simultaneous) two-photon absorption.

First, as we argue in the manuscript (lines 172-177), the power of our continuous wave 633 nm HeNe laser line we are using for the excitation is at least three orders of magnitude lower than peak intensity of pulsed lasers commonly used in two photon absorption microscopy. Furthermore, with a potential two-photon excitation wavelength around 317 nm, one would expect a more blue shifted emission maximum, as 317 nm light is expected to efficiently excite well known and most abundant autofluorophores like NADH and FAD, with broad emission peaks around 460 and 540 nm, respectively. However, we did not observe any emission bellow 560 nm (Fig 3A in the manuscript), making two-photon absorption very unlikely hypothesis to explain the anti-Stokes emission we report.

*3) The authors argue that 590nm emission does not correspond to any know molecules but cite a very old reference (ref 9) which actually says cellular auto-fluorescence in the 500-600 nm spectral region is mostly associated with flavins. Although it’s agreeable that the exact maxima at 590nm is not well known and FAD emission is more blue-shifted from 590nm, but care must be taken to rule out flavins. Flavins are present in diverse form of molecules and oxidation states, especially in mitochondria and fluorescence is extremely sensitive to microenvironment. How many cells did the authors measure to confirm that 590nm emission is a ubiquitous in the mitochondria of HELA cell and there is no spectral shift from 590nm? Why did the authors choose 633nm? Where is the excitation maxima for that 590nm emission, is it 633nm? An excitation spectra would be valuable as it provides clue about the absorption and electronic states of the molecule of interest. Therefore it’s not convincing that authors rule out 2-photon excitation based on the their laser intensity.*

We performed more extensive literature review on emission spectra of various autofluorophores including more recent publications. However, to the best of our knowledge, no autofluorophores (including varying oxidation states) with the emission peak around 590 nm are reported so far.

Even though flavins are most often associated with emission around 500-600 nm, number of other intrinsic and extrinsic molecules (for example cytochromes, lipopigments or phenol red) were reported to be emissive in 550-600 nm range. Furthermore, even NADH or metalloproteins cannot be safely ruled out, as their photophysical properties were reported to be highly environment dependent. In the manuscript we put special care to rule out **extrinsic** fluorophores like phenol red, as its contribution can be easily verified. However, finding the exact type of molecules responsible for anti-Stokes emission reported here becomes very difficult when it comes to **intrinsic** fluorophores. Difficulty comes not only from the sheer number of potential autofluorophores or lack of negative controls, but also complex photophysical behavior depending on configuration, oxidation state or response to specific microenvironment. Furthermore, we believe that more than one species could be responsible for observed anti-Stokes emission as anti-Stokes emission originating in mitochondria also can be observed using 560 nm excitation (Figure 1E), making identification of specific autofluorophores even more complicated.

Therefore, for now in this paper we chose to focus on applications and potential artefacts rather than identify exact type of molecule responsible for this emission.

We did not do an elaborate statistical analysis on spectral shape and peak position of observed emission as it wasn’t the scope of our work and the overall Anti-stokes emission is dim and subject to photobleaching, limiting the quality of the emission spectra and hence the analysis of emission maxima and other spectral properties. However, we measured multiple anti-Stokes emission spectra on live, non-stained HeLa cells, focusing on different cells and their mitochondria. Figure below shows minimal spectral variations between different mitochondria and cells.

It would certainly be beneficial to measure anti-Stokes emission excitation spectrum, lifetime and excitation power dependency to certainly rule out two photon absorption possibility, but it is practically extremely challenging. Photobleaching is the biggest problem, limiting the possibility to measure excitation spectra, lifetimes and excitation power dependencies. We added these arguments to the manuscript.

*4) The authors have randomly thrown in two possible mechanism of hot-band and dark states without any clear argument. Hot band emission should have an intense stokes component in red-region along with the anti-stokes yellow emission. A dark state emission is long-lived and should have a fluorescence lifetime an order higher in magnitude. Did the authors observe any of these?*

When it comes to an origin of observed anti-Stokes emission, multiple photophysical pathways could be considered, including, but not limited to “hot band” excitation of thermally (Boltzmann) populated states, excited state annihilation (singlet or triplet) or consecutive photon absorption through a long lived intermediate state. We also added chemiluminescence (line 171 in the manuscript (1)) as another plausible route.

So far we showed that it’s not likely to be due to two-photon absorption (see previous comments for the arguments). To address “hot band” absorption, we have measured emission on the Stokes side (as suggested by the reviewer) and observed weak, broad and featureless emission spectrum. Even though observed tail of emission spectrum could be a strong argument in case of a sample with single type of fluorophores, it is not conclusive in mitochondria, where multiple autofluorophores have been reported.

Other possible mechanisms could be investigated in more detail by analyzing Stokes and anti-Stokes emission lifetimes. However, as we pinpoint in the previous comment, it is not practically possible to record it due to extremely low fluorescence intensity and fast bleaching.

Since we are physically limited by the number of photons that can be recorded before bleaching, in this paper we only put an effort to show that mechanism responsible for this anti-Stokes emission is different than commonly reported 2-photon absorption.

*5) The data presented in figure 4 is unclear and not explained. Are the scans shown in A-F is from the same ROI? If so, what was the time interval? Why the images looks significantly different from each other? Are the scan:A-D and scan:G-I from same ROI? Why the signal was delocalized, especially if it originates in mitochondria?*

Micrographs in Figure 4 A-F show consecutive scans of live, label-free HeLa cells and represent the same ROI. As we are using APD for detection instead of camera, it takes around 6 minutes to complete the scan. Meanwhile non-fixed, live cells and their organelles were observed to move (S2 Fig. in SI) significantly. We have now specified these parameters in the revised manuscript (highlighted additions at page 10).

G-I scans correspond to different ROI than A-F, but rather show different distribution of anti-Stokes emitting molecules in the cell after cell stress was induced. Multiple reports have shown that cellular response to stress can be investigated following mitochondria dynamics – fission/fusion. As most of the autofluorophores are localized in mitochondria, autofluorescence are often used to evaluate cell stress/viability (2-5). In case of extreme stress - cell death - membrane potential (including mitochondria membrane potential) is lost, membranes become permeable and previously localized molecules become free to diffuse throughout the cell volume. To make these points clearer we have now added more discussion on cell viabilityon page 10 line 202-209.

Figure 4 demonstrates one of the main advantages of the method we are reporting here, showing that cell viability can be followed by looking at anti-Stokes emission. That not only allow investigate label-free cell viability without use of intense UV irradiation but also allows observation of cellular response to stress in stained samples. This wasn’t the case previously as Stokes autofluorescence is always buried under significantly more intense emission of external fluorophore in labeled cells.

*6) Note: ref 4 and ref 12 are same*

References have been corrected.

In summary, the observation presented in the current manuscript is interesting. However the presented data is largely unexplained, looks preliminary and not fit for publication in its current state. Authors are encouraged to perform additional experiments to characterize the phenomena thoroughly, perform an extensive revision of their manuscript in the context of present literature, clearly present the research advancement and finally improve the scientific rigor of the data analysis and discussion for the interest of their future readers.

We share the wish of the reviewer to fully understand the molecular and mechanistic origin of this new emission. However, we also strongly believe that this first observation of the phenomena and its potential applications merits publication, even though these academically interesting questions are not yet resolved. We hope that our reply and changes to the manuscript have satisfactory addressed the reviewers’ questions.

1.	Ciscato LFML, Weiss D, Beckert R, Bastos EL, Bartoloni FH, Baader WJ. Chemiluminescence-based uphill energy conversion. New J Chem. 2011;35(4):773-775.

2.	Westermann B. Bioenergetic role of mitochondrial fusion and fission. Biochimica et Biophysica Acta (BBA) - Bioenergetics. 2012;1817(10):1833-1838.

3.	Youle RJ, van der Bliek AM. Mitochondrial fission, fusion, and stress. Science. 2012;337(6098):1062-1065.

4.	Aubin JE. Autofluorescence of viable cultured mammalian cells. Journal of Histochemistry & Cytochemistry. 1979;27(1):36-43.

5.	Dittmar R, Potier E, van Zandvoort M, Ito K. Assessment of cell viability in three-dimensional scaffolds using cellular auto-fluorescence. Tissue engineering Part C, Methods. 2012;18(3):198-204.
