# Supplementary material for: Intrinsic anti-Stokes emission in living HeLa cells
Source: PLoS One. 2020 Mar 16;15(3):e0230441. doi: 10.1371/journal.pone.0230441 (PMC7075565; doi:10.1371/journal.pone.0230441)
Supplement: Attachment — Submitted filename: Response to Reviewers.docx [file pone.0230441.s007.docx]

**January 7^th^ 2020**

**Laura Kacenauskaite**

PhD fellow

Nano-Science Center &

Department of Chemistry

University of Copenhagen

Universitetsparken 5

DK-2100 Copenhagen Ø

Denmark

Tel: +45 71420031

laura.kacenauskaite@chem.ku.dk

f

Dear Dr Debabrata Goswami and editorial board of *PLOSONE*,

please find attached our answers to the reviewers regarding the manuscript entitled:

**Intrinsic anti-Stokes emission in living HeLa cells,**

*by Laura Kacenauskaite, Dovydas Gabrielaitis, Nicolai Bærentsen, Karen L. Martinez, Tom Vosch and Bo W. Laursen*

We addressed all reviewer comments in detail. Our corresponding changes in the manuscript are marked in yellow, whereas parts where editor and reviewer comments have been addressed are marked in blue.

We hope that with these changes the manuscript can be accepted for publication.

Best regards,

Laura Kacenauskaite

Reply to editor and reviewers:

***Reviewer #2:*** *The authors have partly addressed the comments previously made. Although the manuscript has been improved, the study appears to have limited impact in its current state. The authors argued that the signal is too weak and photo-bleaching too high to perform statistical analysis, lifetime measurement or even to identify the excitation maximum. This argument would raise a doubt about the significance or usefulness of this newly observed anti-stokes emission in this field, where other reported emissions are considerably strong. It would be advisable to take more time to perform additional experiments to characterize the new emission, thus, making the paper more valuable for the readers.*

The main concern of reviewer #2 in this second review is the question of impact or significance of our results based on the argument that if the signal is too weak to measure fluorescence lifetime or excitation spectrum, the signal might not be significant and thus relevant to be investigated.

We disagree with this reasoning. As clearly shown in the data of the manuscript, the mitochondria signal resulting from this new anti-Stokes imaging is unambiguous, reproducible and can even be used to visualize cell stress.

Anti-Stokes emission microscopy using upconverting fluorophores can be used today by anyone with a microscope setup equipped with sensitive avalanche photodiodes and is thus becoming an important and highly studied modality in bioimaging. We believe that it is important to report intrinsic anti-Stokes emission from the HeLa cells in the range that so far was always considered to be completely “background-free”, in particular when this signal is informative by indicating the stress level of the cell.

To emphasize the significance of reported anti-Stokes emission we added the following to the manuscript:

The possibility to exploit anti-Stokes emission to obtain information about the stress level of cells has several advantages. First of all, the signal is intrinsic and does not require the addition of any fluorophores to the live cells. Its detection is furthermore done with distinct optical conditions (in comparison to regular cell dyes) which makes it compatible with many other fluorophores used in cells. It enables the use of one more fluorophore/modality in multi-labeled samples and thereby provides more information about each cell composing the sample.

These features are very timely as anti-Stokes microscopy using upconverting fluorophores is becoming an important and highly studied modality in bioimaging that can be detected with any microscope setup using sensitive avalanche photodiodes.

We share the wish of the reviewer to fully understand the molecular and mechanistic origin of this new emission and we agree that the lifetime and excitation spectra measurements, that reviewer #2 wish for, could be a good complement to our results from the fundamental point of view. However, getting these results requires very different instrumentation and emission intensities than what is required for imaging. We admit that we are not able to obtain these data as we depend on and are physically limited by the instrumentation that is available.

Indeed, to record a reliable excitation spectrum of observed anti-Stokes emission, extremely powerful whitelight laser/lamp is necessary (output power of *at least* 10 mW per excitation width can be estimated based on our results and experience with 633 nm line of HeNe laser). We have previously attempted to record an excitation spectra using SuperK ExtremeEXB-6 with Super K SELECT wavelength selector from NKT Photonics, but no signal was observed due to too low excitation power (less than 1mW per excitation width). SuperK Extreme EXR-20 supercontinuum laser produced by the same company (which to our knowledge is the brightest commercially available whitelight laser on market to this day), also would not be sufficient as it “only” reaches ~3-7 mW/nm. That leaves us with only a few very specialized places in the world where such experiment could be carried out. Same technical limitations, together with fast bleaching, also apply to fluorescence lifetime measurements.

Furthermore, we also do not have hope that recorded excitation spectra could actually help to identify the molecule, let alone the mechanism, responsible for this anti-Stokes emission. As it was previously also noted by the reviewer, difficulty comes not only from the sheer number of potential autofluorophores but also complex photophysical behavior depending on configuration, oxidation state or response to specific microenvironment. Lack of possibilities to prepare and measure reliable negative controls in live cells then demand *ex vivo* ‘deconstruction’ of mitochondria molecule by molecule, which is practically impossible. Fluorescence lifetime measurements maybe could show if triplet state formation is involved in the process, but otherwise also in practice would not be helpful to identify anti-Stokes emitting species.

**Editor:** *The main point of concern of the paper that was raised by one of the referees is the important issue of the statistical significance of the new spectral features and that part is being brushed aside.*

*The authors seem to not interested to address the main concern of one of the reviewers on the lack of data and statistical significance of the work in their revised submission. Concern on the confidence level on new spectral signature need to be justified before the paper may be published.*

The second point raised by both reviewer and the editor concerns statistical significance of our work. In the first review reviewer #2 specified this questions as: “How many cells did the authors measure to confirm that 590nm emission is a ubiquitous in the mitochondria of HELA cell and there is no spectral shift from 590nm”.

To ensure that observed intrinsic anti-Stokes emission is a general property of HeLa cells we imaged multiple cells from different batches under the same conditions. We have in this 2^nd^ revision added further details on the number of cells and cell cultures investigated in the study. The following text has been added (and is highlighted in the submitted manuscript):

The anti-Stokes emission of HeLa cells has been studied on ≈50 cells from 6 independently grown cell cultures throughout the course of half a year. The same signal localized in/on mitochondria has been detected in 100% of the cells imaged and the anti-Stokes emission spectra recorded on 10 % of them (randomly selected) were all peaked at around 590 nm.

While this number of samples and spectra certainty is insufficient to derive any statistical significant quantitative statements e.g. of intensity of the emissions as function of cell conditions, we find it sufficient to support the qualitative scope of the paper: 1) reporting a new anti-Stokes imaging modality for mitochondria in HeLa cells and 2) drawing attention to this emission for other researchers applying imaging modalities for which this emission can interfere.

We hope that our reply and changes to the manuscript have satisfactory addressed the reviewers’ and editors concerns.
