# Supplementary material for: Intrinsic anti-Stokes emission in living HeLa cells
Source: PLoS One. 2020 Mar 16;15(3):e0230441. doi: 10.1371/journal.pone.0230441 (PMC7075565; doi:10.1371/journal.pone.0230441)
Supplement: Attachment — Submitted filename: Response to Reviewers.docx [file pone.0230441.s008.docx]

**February 20^th^ 2020**

**Laura Kacenauskaite**

PhD fellow

Nano-Science Center &

Department of Chemistry

University of Copenhagen

Universitetsparken 5

DK-2100 Copenhagen Ø

Denmark

Tel: +45 71420031

laura.kacenauskaite@chem.ku.dk

f

Dear Dr Debabrata Goswami and editorial board of *PLOS ONE*,

Please find attached our answers to the reviewers regarding the manuscript entitled:

**Intrinsic anti-Stokes emission in living HeLa cells,**

*by Laura Kacenauskaite, Dovydas Gabrielaitis, Nicolai Bærentsen, Karen L. Martinez, Tom Vosch and Bo W. Laursen*

We addressed all reviewer comments in detail. Our corresponding changes in the manuscript are marked in yellow, whereas parts where editor and reviewer comments have been addressed are marked in blue.

We hope that with these changes the manuscript can be accepted for publication.

Best regards,

Laura Kacenauskaite

Reply to editor and reviewers:

Reviewer #2: The authors have improved the manuscript in this version. With the following minor revisions, it may be recommended for publication and further review may not be required.

We are grateful for Reviewer’s#2 thorough suggestions and comments towards inproved manuscript. Reviewer comments were considered in detail and addressed bellow:

1) It seems that the authors are physically limited by avilable facility, which often happens with scientific studies. However, if those inteded experiments can reveal imprtant clue about the emission, it needs to be discussed in the manuscript as a limitaion of the current study and possible future experiments for others in the field.

We have briefly mentioned the current limitations for further spectoscopic measurements in lines 190-191 and added more details in the revised manuscript.

2) Why 4C, 15m was chosen for cell stress experiment may be briefly explained in the result/method section.

We added a short explanation on cell stress in Materials and methods section.

We hope that our reply and changes have satisfactory addressed the reviewers’ comments and manuscript can now be accepted for publication.
